# Response Surface Methodology Optimization of Resistance Welding Process for Unidirectional Carbon Fiber/PPS Composites

**DOI:** 10.3390/ma17102176

**Published:** 2024-05-07

**Authors:** Da-Wei Yu, Xiao-Ting Qing, Hong-Yu Lin, Jie Yang, Jia-Cao Yang, Xiao-Jun Wang

**Affiliations:** 1 College of Polymer Science and Engineering, Sichuan University, Chengdu 610065, China; yudawei0515@163.com (D.-W.Y.); qingxt@stu.scu.edu.cn (X.-T.Q.); linhy@stu.scu.edu.cn (H.-Y.L.); 2 State Key Laboratory of Polymer Materials Engineering, Sichuan University, Chengdu 610065, China; ppsf@scu.edu.cn; 3 Institute of Materials Science and Technology, Analysis and Testing Center, Sichuan University, Chengdu 610065, China

**Keywords:** resistance welding, UCF/PPS, response surface method (RSM), lap shear strength (LSS), optimal process parameters

## Abstract

The use of thermoplastic composites (TPCs) as one of the lightweight solutions will inevitably encounter problems in connection. Resistance welding has the characteristics of high strength, simplicity, and high reliability, and is considered a very potential hot-melt connection technology. The resistance welding technology of unidirectional carbon fiber-reinforced polyphenylene sulfide composites (UCF/PPS) was systematically studied. The experimental results show that the 100-mesh brass mesh has the best resin wetting effect and heating efficiency, and the PPS/oxidized 100-mesh brass mesh composite resistance element (Ox-RE/PPS) has the highest welding strength. The welding failure mode changes from interface failure and RE failure to interlayer structure damage and fiber fracture. The single-factor experimental results show that the maximum welding strength is reached at 310 °C, 1.15 MPa, and 120 kW/m^2^. According to the conclusion of the single-factor experiment, the Box–Behnken method was further used to design a three-factor, three-level experiment, and a quadratic regression model was established according to the test results. The results of variance analysis, fitting curve analysis, and perturbation plot analysis proved that the model had high fitting and prediction abilities. From the 3D surface diagram analysis, the influence of power density is the largest, and the interaction between welding temperature and power density is the most significant. Combined with the analysis of Design Expert 13 software, the optimal range of process parameters was obtained as follows: welding temperature 313–314 °C, welding pressure 1.04–1.2 MPa, and power density 124–128 kW/m^2^. The average strength of resistance welding joints prepared in the optimal range of process parameters was 13.58 MPa.

## 1. Introduction

TPCs are composites composed of thermoplastic resins and various different materials combined by physical or chemical processes [1,2]. The strength of the material can be enhanced, and the economic benefits can be improved, by utilizing various combinations of reinforcement materials. TPCs have excellent mechanical properties, as well as being lightweight, rapid prototyping, easy repair, and having strong design ability [3,4]. With the research of many scholars in recent years, the composite materials of high-performance thermoplastic resins, such as polyphenylene sulfide (PPS), polyetheretherketone (PEEK), polyetheretherketone ketone (PEKK), and polyetheretherimide (PEI), have developed rapidly [5,6,7]. TPCs are replacing thermosetting composites (TSCs) in defense, military industry, aerospace, rail transportation, civil, and other fields [8]. However, the increase in usage and the expansion of market share also bring a series of problems and challenges, such as the connection methods of TPC components [1,9]. At present, high-performance thermoplastic resin composites are mainly used in secondary load-bearing structures and some primary load-bearing structures. It is of great significance to explore ways to improve joint performance and find solutions that meet the connection standards of primary load-bearing structures to promote innovation and progress in the field of industrial manufacturing.

Nowadays, the most mature connection methods are still the traditional connection methods, such as mechanical connection and bonding [10,11]. The disadvantage of the former is that the use of rivets or bolts can cause local stress concentration in the parts or cause defects in the composite material. The disadvantage of the latter is that it requires high pretreatment of the bonding surface, and the performance of the bonding is greatly affected by the environment. To address the mismatch between the development of connection technology and materials, it is necessary to explore effective and reliable methods of connection. According to the current practical application needs, appropriate connection methods should be selected to ensure the reliability and convenience of composite part connections [12,13]. Due to the remelting property of the thermoplastic resin matrix, TPCs can be heated for shape change and retreatment [14]. Therefore, in addition to traditional connection methods, hot-melt connection technology can also be used to connect. Hot-melt connection technology contains many subcategories, but the three most promising ones are resistance welding (RW), induction welding (IW), and ultrasonic welding (UW) [15,16,17,18,19]. These three technologies solve problems that are difficult to solve with traditional techniques, such as automation, controllability, high strength, etc., and can be applied to different application scenarios [20,21].

The resistance welding method studied in this paper has the characteristics of being simple and flexible, a short process, and having no surface treatment. It is an economical and efficient welding method and has great potential in the field of TPC connections. The resistance welding process takes about a few minutes. The resistance element (RE) in the welding layer is energized to generate heat, thereby increasing the temperature of the joint. The resistive elements can be carbon fiber, brass mesh, stainless steel (SS) mesh, or conductive nanocomposites [22,23]. The welding layer can also be innovatively mixed with carbon nanotubes, which enhances the welding interface [24]. Marti et al. [25] investigated the availability of different sandwich-type heating components for enhanced resistance welding. Most thermoplastic resins were easy to crystallize, and the cooling rate during resistance welding had a significant impact on the crystallinity and morphology [26,27,28]. Liu et al. [29] studied the relationship between geometric size, current density, and temperature response in the self-resistance electric (SRE) heating method of composite materials; determined the relationship between temperature rise rate and current density; and explored the deconsolidation behavior of SRE after heating. The final effect of resistance welding of thermoplastic composites also depended on the regulation of various process parameters, including welding current, clamping pressure, heating time, etc. [30]. Xiong et al. [31] used carbon fiber felt (CFF) composite film instead of pure resin film as a fusible component of electric resistance welding. The addition of CFF improved the lap shear strength (LSS) of welded joints and shortened the welding time (tw). LSS refers to the shear force that the unit welded surface can withstand when the welded surface is damaged, and its unit is expressed in megapascals (MPa). Barbosa et al. [32] used means of fracture analysis to study the resistance-welded joints of composite laminates. They believed that the identification of fracture morphology provides information about failure modes and contributes to the quality of the welding process. Under the same welding time and electrode pressure, as the welding current increases, the failure mode of resistance element welding (REW) joints changes from interface mode to partial button fracture [33].

With the continuous development of computer science, the welding process can be simulated and optimized with the help of computers and software [23]. Lionetto et al. [34] accurately modeled the temperature distribution of laminates in continuous induction welding of thermoplastic matrix composites. Machine learning can also be used for multi-objective optimization of complex welding processes to achieve an optimal solution [35]. Box–Behnken design (BBD) is a design method of response surface experiment that is commonly used to study the influence of multiple factors on a response variable [36,37,38]. Using Design Expert 13 software, we can determine the nonlinear relationships between factors through a small number of experiments and establish corresponding mathematical models. Vinayagamoorthy R optimized the drilling parameters of composite materials using the Box–Behnken method, as well as each output factor, and finally obtained the best processing conditions [39].

Polyphenylene sulfide (PPS) is a high-performance engineering plastic with excellent mechanical properties, heat resistance, chemical stability, and electrical insulation. However, due to the high crystallinity and low surface energy of PPS resin, there are some limitations when using traditional methods for connection. Its unidirectional carbon fiber-reinforced polyphenylene sulfide composite (UCF/PPS) is one of the most important thermoplastic composites. Therefore, it is of representative significance to study the resistance welding process of UCF/PPS. Generally, there are many factors that affect the final welding effect of UCF/PPS resistance welding. Therefore, in order to achieve efficient and stable welding effects, resistance welding technology needs to be systematically explored and improved. In this study, the effects of resistance element mesh size, processing methods, pressure holding time, welding temperature, welding pressure, and power density on welding strength were evaluated. This paper proposes the use of the Box–Behnken design response surface method (BBD-RSM) to optimize welding parameters. The response surface curve was drawn to evaluate the influence of three main process factors on welding quality, and the interaction between the factors was studied. The corresponding quadratic regression model was established to optimize the welding conditions and summarize the best process parameter range. Finally, the accuracy of the quadratic regression model prediction was verified by practice.

## 2. Materials and Methods

### 2.1. Materials

The materials used for the resistant welding experiments in this research were UCF/PPS composite materials (Nanjing Advanced Thermoplastic Composite Co., Ltd., Nanjing, Jiangsu, China). The UCF/PPS was a unidirectional carbon fiber prepreg with a nominal fiber volume content of 68%, a thickness of 0.3 mm, and a width of 90 mm.

**Figure 1:**
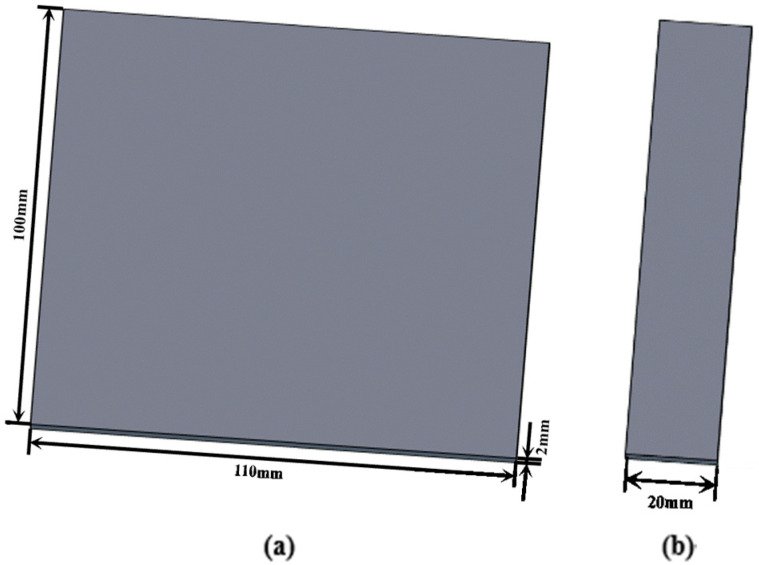
(**a**) Schematic diagram of UCF/PPS laminate and (**b**) schematic diagram of UCF/PPS specimen.

The UCF/PPS prepreg was pressed into a laminate with a thickness of 2 mm, a width of 100 mm, and a length of 110 mm, as shown in Figure 1a. The stacking direction of the laminates was unidirectional, with a total of nine layers. Specimens with a length of 100 mm and a width of 20 mm were cut from the laminates, as shown in Figure 1b.

PPS film was made of PPS resin pellets (Xinjiang Zhongtai Chemical Co., Ltd., Urumqi, Xingjiang, China). Its melting and initial decomposition temperatures were 283 °C and 430 °C, respectively. PPS resin was pressed into films with a thickness of 0.1 mm using a hot press at 300 °C. After cooling, the slices were cut into a length of 25 mm and a weight of 20 mm for use.

In this study, a 40-, 100-, and 200-mesh brass mesh was selected as the resistance element (RE). The brass (international standard name: CuZn35) plain weave meshes were provided by Anping County Mujie Wire Mesh Products Co., Ltd., Anping, Hebei, China.

### 2.2. Preparation of the Resistance Element

Resistance welding of thermoplastic composite materials uses the Joule heating effect to heat and melt the resin to join two specimens together. The power density per unit area in the welded joint is adjusted to control the heat and temperature distribution during welding. The power density can be calculated using the following formula [40]:

*P = IU/LW* (1)

In the formula, *P* is the power density in kW/m^2^, *I* is the current in A, *U* is the voltage in V, *L* is the welding length in mm, and *W* is the welding width in mm.

**Figure 2:**
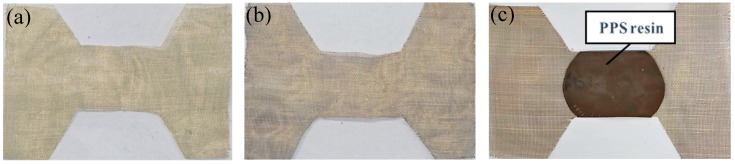
Resistance elements: (**a**) UT-RE, (**b**) Ox-RE, and (**c**) Ox-RE/PPS.

In this research, brass resistance elements were cut to the same dimensions. The resistance elements were cleaned using ethanol and deionized water, and then they were dried for future use, as shown in Figure 2a. For high-quality welded joints, oxidation treatment and the addition of resin layers were used in this study to optimize the untreated resistance elements (UT-RE). When exploring different parameters, the high strength of the joint can make the difference more obvious. The resistance element was oxidized (Ox-RE) by placing it in a muffle furnace at 350 °C for 30 min, as shown in Figure 2b. The welding area of the resistance element was sandwiched between two PPS resin films and placed on a constant-temperature heating platform at 320 °C. PPS resin composite resistance element (Ox-RE/PPS) was prepared using the hot pressing method, and the thickness of the resin layer was controlled to 0.3 ± 0.02 mm, as shown in Figure 2c.

### 2.3. Experimental Methods

#### 2.3.1. Resistance Welding Experiment

(1) The single-lap resistance welding experiment

**Figure 3:**
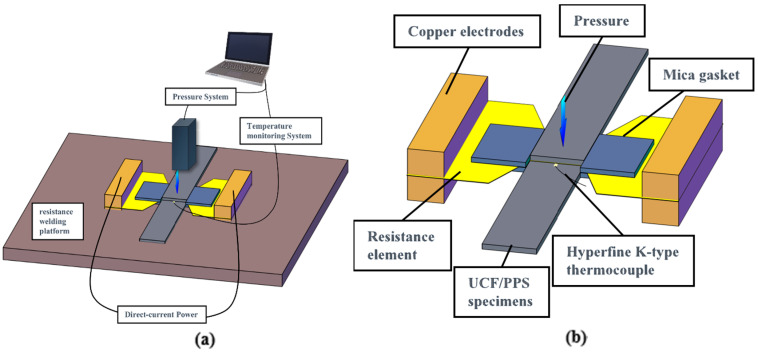
(**a**) Single-lap resistance welding experimental device and (**b**) schematic diagram of PPS/UCF single-lap resistance welding.

As shown in Figure 3a, the single-lap resistance welding experimental device consisted of a direct-current (DC) power supply, temperature monitoring system, pressure system, and resistance welding platform. The DC power supply (DP5060, Mestek Tools Co., Ltd., Shenzhen, China) was connected to the resistance element via copper electrodes, providing a maximum output power of 3000 W. The temperature monitoring system (YET-640X, Suma Electric Instrument Co., Ltd., Xinghua, China) adopted a hyperfine K-type thermocouple with a diameter of 0.1 mm. The thermocouple probe was wrapped with insulating tape to monitor and record the temperature inside the welding layer in real time. A universal testing machine (model E45, MTS Industrial Systems, Shenzhen, China) and a custom epoxy pressure block were used for constant displacement pressurization during welding.

The specimens, resistance element, and hyperfine K-type thermocouple were placed on the welding platform and fixed with a horizontal fixture, as shown in Figure 3b. The temperature measurement point was located at the center of the welding layer, and the thermocouple was removed when heated to the target temperature. Mica gaskets were placed on both sides of the sample joint to control the thickness and limit the deformation of the sample during welding. The mica gasket also played a role in preventing excessive loss of the molten resin under pressure, thus reducing the production of defects.

(2) The operation procedure for resistance welding

The operation procedure for resistance welding is described as follows. Before the experiment, the welding surface was polished with 320-mesh sandpaper, cleaned with absolute alcohol, and dried in the oven. The universal testing machine was controlled to press the welding part to avoid heat loss. The power was turned on, and the welding layer temperature began to rise. When the temperature of the welding layer reached 150 °C, the pressure was adjusted to the experimental value to ensure that the initial pressure was consistent for each test. Heating was continued to the target temperature, and then the power was turned off. Before taking out the sample, the pressure was maintained for a period of time.

#### 2.3.2. Single-Factor Experimental Design

Prior to the formal resistance welding experiments, the resistance welding conditions of UCF/PPS composite specimens were initially explored to determine the approximate range of the studied parameters. The mesh size and treatment method of the resistance element used in the welding experiment directly affect the strength of the welded joint. In addition, PPS resin undergoes the process of melting and reconsolidation during welding, and the degree of infiltration, melting, and crystallinity of the resin are affected by the pressure holding time, welding temperature, welding pressure, and power density [30]. The welding temperature affects the fluidity of the resin. When the welding power density is constant, the welding temperature increases with the increase in welding time. Therefore, the welding temperature, welding time, and total power density are regarded as equivalent process parameters in this study [41].

The above six single factors are studied to determine the best process range of each factor and the main process parameters of response surface design. The mesh sizes of the resistance elements are 40, 100, and 200 mesh, respectively. The RE process methods include untreated, oxidation treatment, and adding PPS resin. The welding process is divided into a pressure holding time of 0–180 s, a welding temperature of 300–330 °C, a welding pressure of 0.55–1.45 MPa, and a power density of 100–160 kW/m^2^.

#### 2.3.3. Optimization of the Resistance Welding Process Using Response Surface Methodology

Under the premise of the best other secondary factors, Box–Behnken design (BBD) with response surface methodology (RSM) was used to optimize the resistance welding process using Design-Expert 13 software. Ox-RE/PPS was selected for BBD experiments. Three main process parameters (A: welding temperature, B: welding pressure, and C: power density) were optimized and adjusted by the response surface method to obtain the best joint strength. The range of values of the three main parameters in BBD is shown in Table 1.

Through the experimental results, the influence factors (i.e., welding temperature, welding pressure, and power density) and their interaction on the lap shear strength (LSS) can be determined. Analysis of variance (ANOVA) was used to determine the degree of influence, significance, and adequacy of the quadratic regression model. Design-Expert 13 software and Origin 2022 software were used to draw 2D array diagrams and 3D response surface diagrams, which intuitively reflected the main influence and interactive influence of influencing factors on the LSS. Finally, based on the response surface model, the joint strength after process optimization was predicted and verified.

### 2.4. Characterization

The test instrument was the universal testing machine (Model E45, MTS Industrial System, Shanghai, China), and the welding quality was evaluated by testing the lap shear strength (LSS) of UCF/PPS welding samples. The lap shear strength refers to the shear force that the unit welded surface can withstand when the welded surface is damaged, and its unit is expressed in megapascals (MPa). The test standard for LSS refers to ASTM D1002 [42], using a 100 kN force transducer without an extensiometer. The dimensions of the weld surface were 20 × 25 mm, the clamping area was 20 × 40 mm, the sample spacing was 75 mm, and the loading rate was 2 mm/min. Five samples were tested in each group.

The failed welding surface was cut into 5 × 5 mm, sprayed with gold, and observed using a scanning electron microscope (SEM, Quanta 250, FEI Corporation, Hillsboro, OR, USA). In the standard test environment, the damage morphology and cross-section of the welding surface were observed using an accelerating voltage of 5.00 kV, an SE imaging mode, and a working distance of 5.6 mm. The failure mechanism of the joints was analyzed based on the macroscopic and microscopic morphology of the fracture.

## 3. Results and Discussion

### 3.1. Effect of Resistance Elements on Welded Joints

#### 3.1.1. Mesh Size

**Figure 4:**
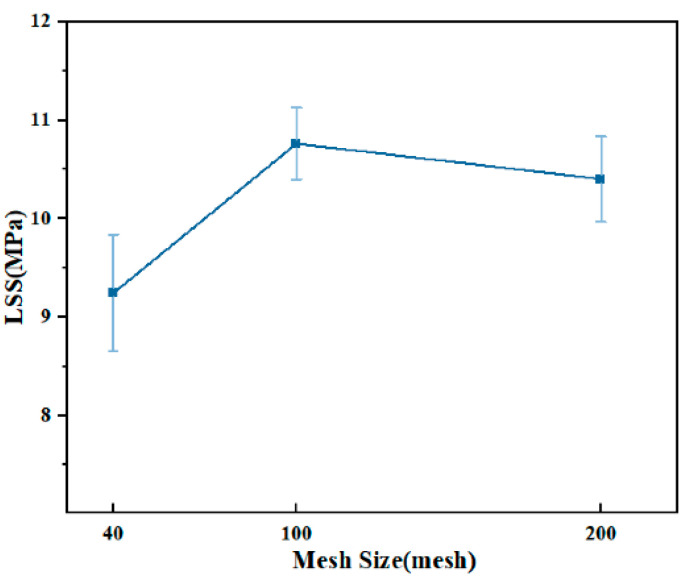
LSS of joints at different mesh sizes.

The resistance element is made of brass mesh with different mesh sizes, which has good electrical conductivity and ductility. The results of the resistance welding experiment are shown in Figure 4. Under the same process conditions, the highest LSS (10.76 ± 0.36 MPa) was obtained by using 100-mesh brass mesh. LSS was significantly lower than in other situations when 40-mesh brass mesh was used. The increase in the mesh number will increase the contact area with the resin, which is conducive to improving the joint strength [43]. However, the mesh pore size will decrease as the number of grids increases. PPS resin is difficult to infiltrate the welding layer, and the strength of the welded joint of the 200-mesh resistance unit is slightly lower than that of the 100-mesh resistance unit.

**Figure 5:**
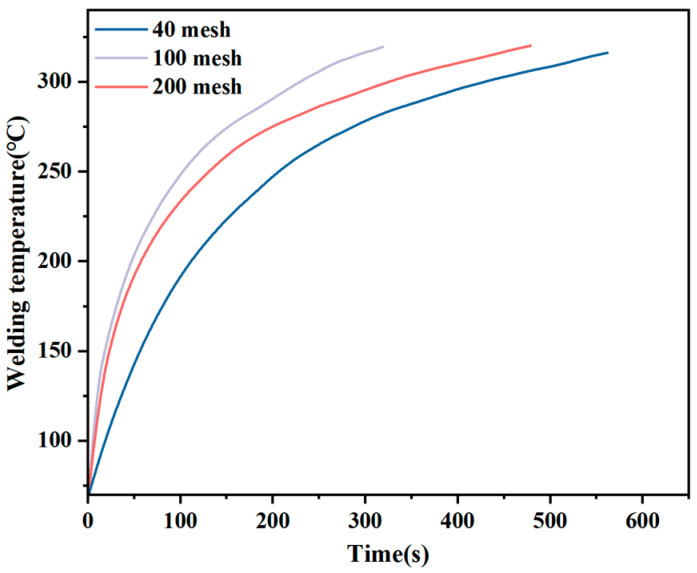
Heating curve: (**a**) brass mesh 40 mesh, (**b**) brass mesh 100 mesh, and (**c**) brass mesh 200 mesh.

During the resistance welding of the UCF/PPS composite, a constant current was applied to the resistive element. The resistance element is energized to produce Joule heating, and the joint starts to heat from the inner layer to the outer layer. The heating curve at the center of the welding layer is shown in Figure 5. Different mesh sizes will directly affect the heat generated during welding [40]. When the number of meshes is small, the heat is concentrated near the wires, and the thermal influence range of each wire is limited. However, the larger the mesh number, the more metal wires in the unit area, and the smaller the total resistance, resulting in less heat being produced. The resistance and thermal influence range of the 100-mesh resistance element is between 40 and 200 mesh, so the test shows that its thermal efficiency is the highest.

**Figure 6:**
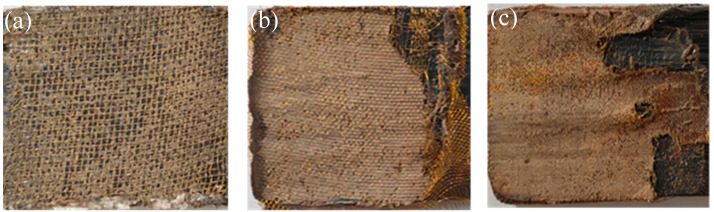
Macroscopic morphology of the failure surface: (**a**) brass mesh 40 mesh, (**b**) brass mesh 100 mesh, and (**c**) brass mesh 200 mesh.

The macroscopic morphology of the failure surface of welded joints with different mesh sizes is shown in Figure 6. After applying a load to destroy these welded joints, it can be seen that the failure occurs at the resin and metal mesh. Among them, due to the poor infiltration of the 40-mesh metal mesh by resin, a large loss of resin in the mesh can be observed. However, the combination degree of 100-mesh and 200-mesh metal mesh with resin is improved. When the joint is damaged, the metal mesh is torn irregularly, which plays the role of the reinforcing phase. In conclusion, 100-mesh brass mesh is the best choice as a resistance element.

#### 3.1.2. RE Process Method

**Figure 7:**
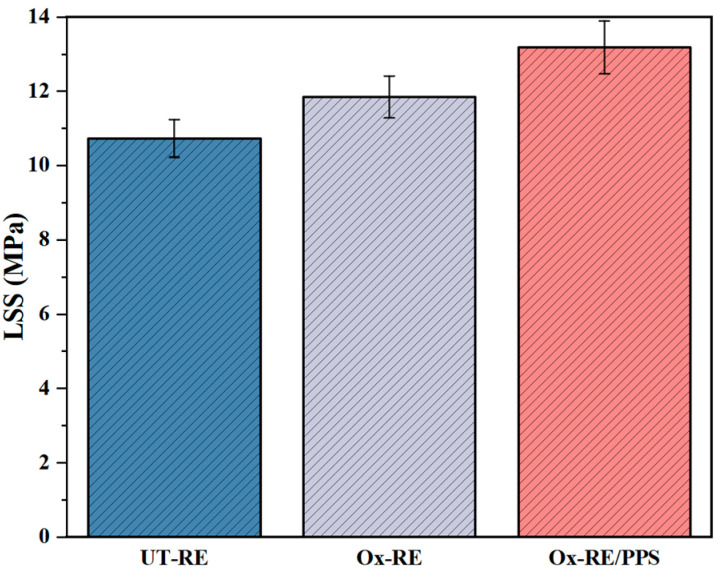
LSS of joints at different RE process methods.

Three types of resistance element, namely, UT-RE, Ox-RE, and Ox-RE/PPS, were used for resistance welding, respectively, and the joint quality was evaluated through the LSS test. The results are shown in Figure 7. The average LSS of the welded joints of UT-RE is 10.73 MPa, and the strength increased to 11.85 MPa after oxidation.

The PPS resin can be fully infiltrated onto the surface of the metal mesh by hot pressing. During the welding process, the resin layer also plays a role in reducing the heat loss in the heating area and making the heat transfer more uniform. The joint strength of Ox-RE/PPS reached 13.18 MPa, which was 11.22% higher than that of Ox-RE. Therefore, more reliable welded joints can be obtained with Ox-RE/PPS compared to UT-RE.

**Figure 8:**
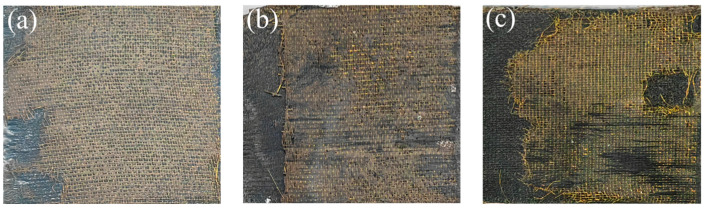
Macroscopic morphology of the failure surface: (**a**) UT-RE, (**b**) Ox-RE, and (**c**) Ox-RE/PPS.

The macroscopic morphology of the failure surface of the welded joint with different RE process methods is shown in Figure 8. Due to the low surface energy of the PPS resin, the adhesion to the brass surface is poor. After high-temperature oxidation of brass, the oxide forms a rough oxide layer on the surface, which helps to improve the surface energy. The PPS resin film and the resistance element were combined by hot pressing. After adding the resin, it can be seen that a small amount of fiber fracture occurs on the failure surface of the joint. It indicates that the resin in the welding layer is immersed in the fibers on the surface of the laminate, so the strength of the welded joint is improved.

**Figure 9:**
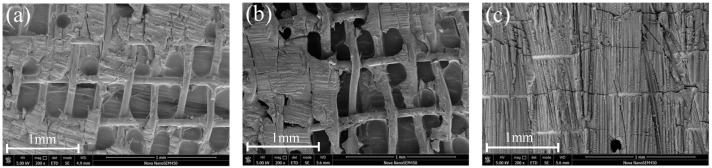
Microscopic morphology of the failure surface: (**a**) UT-RE, (**b**) Ox-RE, and (**c**) Ox-RE/PPS.

The SEM in Figure 9a shows that the adhesion between the surface of the untreated brass wire and the resin is poor, and the surface of the wire is smooth with only a small amount of resin remaining. Failure occurs at the brass wire/resin interface failure and resin/matrix debonding. The microscopic morphology of the Ox-RE welded joint is shown in Figure 9b. After the joint was damaged, more resin remained on the surface of the wire, showing a tearing state. The microscopic morphology of the Ox-RE/PPS welded joint is shown in Figure 9c. Interlayer failures include fiber breakage and resin failure. After the damage, the brass mesh was still largely coated with resin.

**Figure 10:**
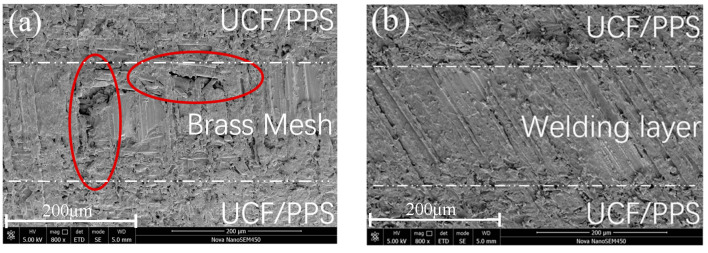
Microscopic morphology of the section: (**a**) Ox-RE and (**b**) Ox-RE/PPS.

SEM was used to observe the microscopic morphology of the cross-sections of the welded specimens (Figure 10a,b). Without the addition of PPS resin, the resin of the UCF/PPS matrix was insufficient to fill the welding layer. In the SEM image, it can be observed that there are obvious pores in the cross-section of the welded joint, which is the weak point inside the joint. After welding using Ox-RE/PPS, all parts of the welded joint were tightly bonded, with good resin infiltration and no obvious defects.

### 3.2. Effect of the Welding Process on the Welded Joint

#### 3.2.1. Pressure Holding Time

**Figure 11:**
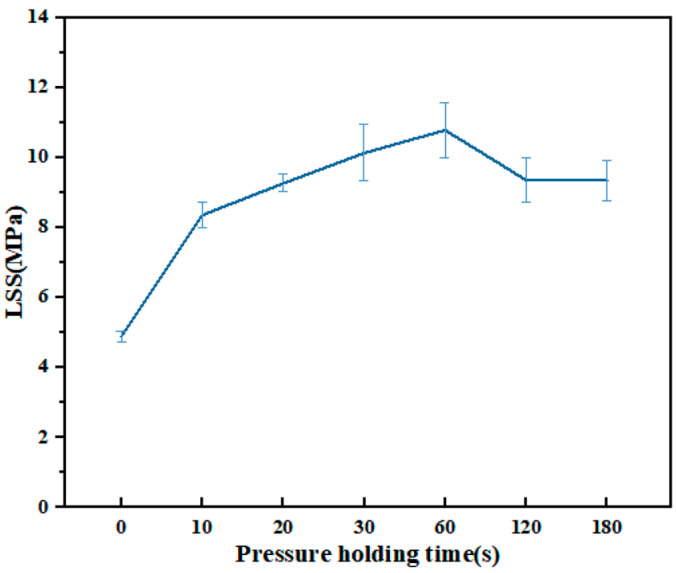
LSS of joints at different pressure holding times.

After the power is turned off, the pressure holding can reduce the cooling rate of the joint, maintain the stability of the joint shape, and reduce the residual stress. PPS resin is easy to crystallize during cooling [26]. Appropriate cooling rates contribute to the formation of an ordered molecular chain structure and improve the mechanical properties, heat resistance, and dimensional stability of the joint. According to Figure 11, the LSS of the joint increases with the extension of the holding time and reaches its maximum at 60 s. The crystallinity of PPS resin is higher when the holding time is more than 60 s, which leads to an increase in joint brittleness. The pressure holding time continues to extend, and the quality of the joint tends to be constant.

#### 3.2.2. Welding Temperature

**Figure 12:**
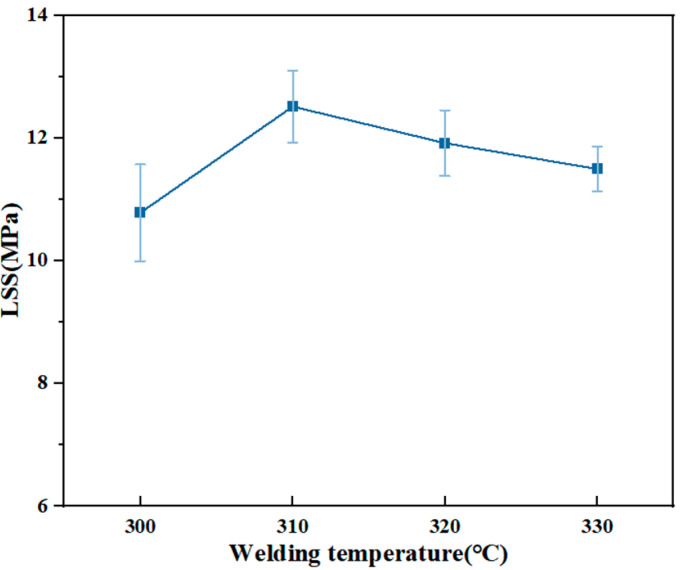
LSS of joints at different welding temperatures.

During welding, the metal wire makes the welding layer resin and the matrix resin melt, and the molecular chain moves to form a whole structure. As shown in Figure 12, with the increase in the highest temperature, the LSS curve shows a trend of first increasing and then decreasing, reaching its maximum value at 310 °C. The edge effect of the resistive element [44] can cause the heating rate at both ends of the welded joint to be higher than that in the middle. Setting a low welding target temperature will result in too little welding time. The resin did not melt sufficiently to form an unstable joint, so the joint strength was not high. In the appropriate temperature range, the higher the welding temperature, the deeper the thermal influence depth of the resistance element in the vertical direction. A too-high welding temperature can significantly improve the mobility of PPS, and the resin is more likely to extrude at the edge of the joint, leading to a decrease in the joint strength. The LSS curve has obvious fluctuations, which can prove that temperature is the main influencing factor during resistance welding. Thermal degradation of PPS resin would be inevitable if the temperature continued to increase [45].

#### 3.2.3. Welding Pressure

**Figure 13:**
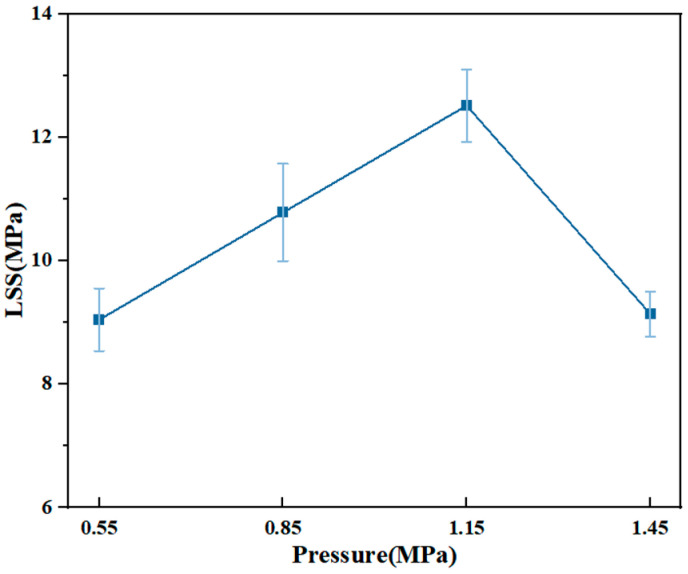
LSS of joints at different welding pressures.

The corresponding welding strength under different welding pressures is shown in Figure 13. Welding pressure is an important factor in resistance welding, which must be applied appropriately to ensure that the spline welding surface is tightly bonded to the welding layer after melting [46]. As the pressure increases, the welding strength first slightly decreases and then increases. At low pressure, the layers of the welded joint are separated and cannot form a good contact. Because of the presence of an air layer, heat cannot be efficiently transferred to the matrix, and the time to complete welding becomes longer during the experiment. Insufficient pressure causes insufficient resin flow in the welding layer and poor joint performance. The optimum welding pressure for the joint is about 1.15 MPa. When the welding pressure increased to 1.45 MPa, the resin at the welding interface was lost under the pressure, and the strength decreased significantly.

#### 3.2.4. Power Density

**Figure 14:**
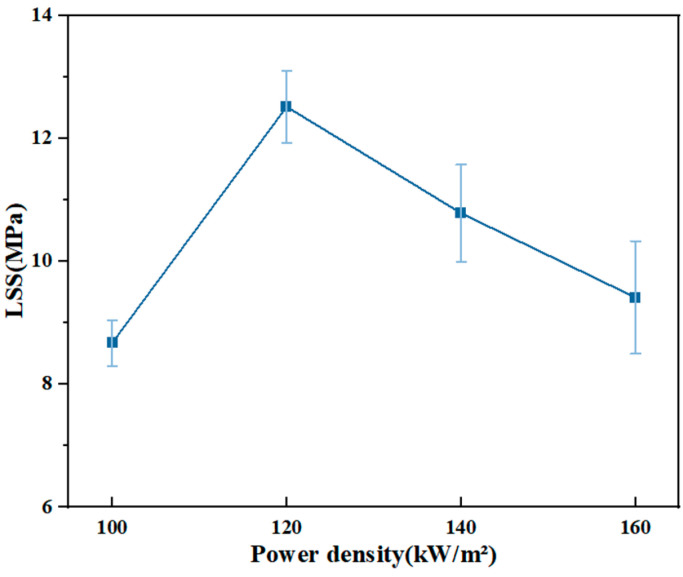
LSS of joints at different power densities.

Preliminary experiments show that a too-low power density cannot form an effective joint. The heating rate increases with increasing power [29]. As shown in Figure 14, the LSS curve shows a trend of first increasing and then decreasing, and the joint strength is the highest at 120 kW/m^2^. At 100 kW/m^2^, the heat generated per unit time is limited, and the fusion of each part of the welding joint is poor, which affects the consistency of the welded joint. The long welding processing time will also increase the economic cost, which is not conducive to the actual processing. High power density can lead to heat accumulation in the joint, which leads to matrix deformation and high-temperature fracture of PPS resin molecular chains, thus reducing the joint strength.

### 3.3. Optimization of the Resistance Welding Process Using RSM

#### 3.3.1. Quadratic Regression Model

Table 2 shows the experimental design based on the Box–Behnken method and its experimental results, which include 17 sets of experiments combined by three independent factors.

The quadratic regression model of welding strength using the Box–Behnken experiment design was based on the uncoded levels of the three independent variables. In this study, the regression equation for resistance welding joints is as follows:

*Y* = −985.75109 + 6.19328 × *A* − 3.52528 × *B* + 0.497637 × *C* + 0.05 × *A* × *B* + 0.002688 × *A* × *C* − 0.0125 × *B* × *C* − 0.010508 × *A*^2^ − 4.86944 × *B*^2^ − 0.005289 × *C*^2^ (2)

In the formula, *Y* represents the LSS in MPa, *A* represents the welding temperature in °C, *B* represents the welding pressure in MPa, and *C* represents the power density in kW/m^2^.

The ANOVA results of the quadratic regression model are summarized in Table 3. The statistical significance and adequacy of the model were verified by variance analysis. The F statistic is the ratio of the regression variance to the error variance and is used to test whether the fit of the regression model is significant. A higher F-value indicates a better fit of the regression model. The *p*-value is the probability of the F statistic and is used to determine whether the F statistic is significant. When the *p* value is less than 0.05, it indicates that the regression model has a significant fit (**). In contrast, when the *p*-value is greater than 1, the fitting degree of the model term is not significant. The coefficient of determination (R^2^) reflects the proportion of the regression model that explains the degree of variation in the dependent variable, with values ranging from 0 to 1. The closer the model is to 1, the better the model fits the observed data.

The F-value of the quadratic regression model is 64.58, and the *p*-value is less than 0.0001, indicating that the model can significantly predict the welding strength. The good fit of the quadratic regression model is contradicted by the fact that the model lacks fit [36]. The F-value of lack of fit in the table is 5.19, and the *p*-value is greater than 0.05, which is not significant, indicating that the model does not lack fit. The coefficient of determination R^2^ is 0.9881, which indicates that 98.881% of the variability in welding strength is explained by this model. This shows that the predicted welding strength is close to the BBD experimental value [37], which confirms the reliability of the model.

**Figure 15:**
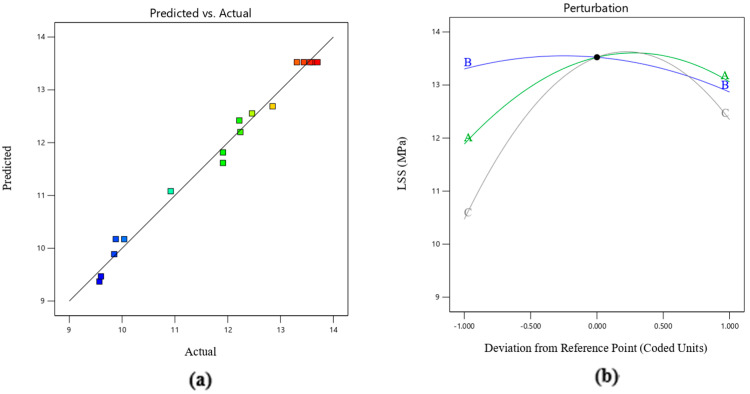
(**a**) Experimental and predicted values of welding strength and (**b**) perturbation plot of welding strength. The color square in Figure 15a correspond to the LSS experiment results in Table 2.

Adeq Precision is the ratio of effective signal to noise, and greater than 4 is reasonable [37]. The precision of this experiment is 21.2956, which indicates that the model has high accuracy in the design space [38]. The experimental and predicted values of welding strength are shown in Figure 15. The difference between the actual value and the predicted value is less than 0.2, indicating that the model has reasonable consistency. In addition, the coefficient of variation (C.V.) was low (2.15%), indicating that the experimental data are accurate and reliable [47].

The *p*-value is used to test the significance level of model items. If *p* < 0.05, it means that there is a statistically significant difference in the item; otherwise, there is no significance [48]. As shown in Table 3, in the established model, A, B, C, AC, A^2^, B^2^, and C^2^ have significant impacts on the model, while other terms have no significant impacts. A perturbation plot of welding strength is shown in Figure 15b. The curvature of the perturbation diagram can reflect the sensitivity of each parameter to the welding strength, and the steeper the slope, the more significant the influence. It can be seen from the figure that the order of influence of the three parameters on LSS is C > A > B, that is, power density > welding temperature > welding pressure.

#### 3.3.2. Effect of Process Parameters on Welding Strength

**Figure 16:**
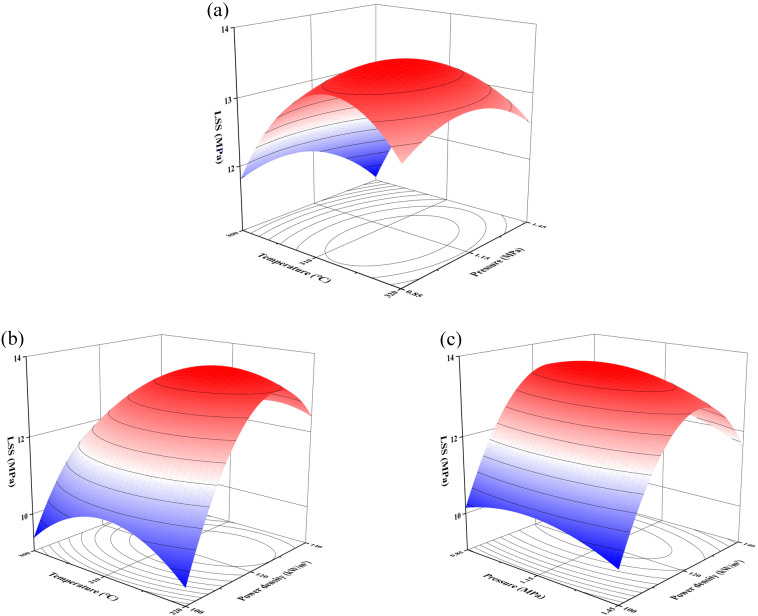
3D surface diagram of interaction: (**a**) T and F, (**b**) T and P, and (**c**) F and P. The welding strength from low to high corresponds to the color in figure form blur to red.

Drawing a three-dimensional surface diagram of the interaction of parameters can more intuitively reflect the influence of parameters on LSS and the interaction between various process parameters (Figure 16). The interaction of T and F was studied by fixing P at 1.15 MPa. Figure 16a shows the synergistic effect of T and F on the welding strength. The TF surface plot is closest to a sphere, proving that the interaction is weak. The analysis shows that the LSS curve fluctuates obviously with the increase in welding temperature under a certain welding pressure, which proves that the temperature is the main influencing factor in the process of resistance welding. During welding, heat is transferred from the inside to the outside. As the temperature of the inner layer increases, the sandwich structure (substrate/resistance element/substrate) is gradually welded together. Excessive temperature can increase the fluidity of PPS and cause deformation of the joint, leading to a decrease in welding strength. When the pressure changes at the same target temperature, the LSS profile curve is relatively straight. This means that at any temperature, pressure plays a role. Low temperatures have a greater impact on pressure than high temperatures.

Figure 16b shows the 3D surface diagram of the influence of a welding temperature of 300–320 °C and a power density of 100–140 kW/m^2^ on welding strength. The power density determines the heating rate during welding, and the target temperature determines the welding time. The TP surface plot is closest to an ellipse, proving that it has the most perfect interaction. Thus, the AC term had a significant effect, and there was a clear interaction. It can be observed that at the same temperature, the slope of the medium and low power density is larger, which has an extremely significant effect on the welding strength. At the same power, the contour of the surface fluctuates obviously, so the power also has an obvious influence on the temperature.

Figure 16c shows the 3D surface plot of the influence of F and P on the welding strength, where the welding pressure is 0.85–1.45 MPa and the power density is 100–140 kW/m^2^. It can be seen from the figure that the curvature of the pressure curve is small for a certain power density. It can be concluded that the influence of welding strength is limited, and the overall curve profile shows a downward trend. When the pressure is the same, the welding strength changes greatly with the increase in pressure. This phenomenon matches the conclusion of the order of influence in the perturbation map.

The variation trend of the 3D surface plot contour showed that the interaction between T and P was the most significant, and the effect of temperature and power on LSS was greater than that of pressure, which was consistent with the results of the ANOVA.

### 3.4. Optimization and Verification of the Model

**Figure A1:**
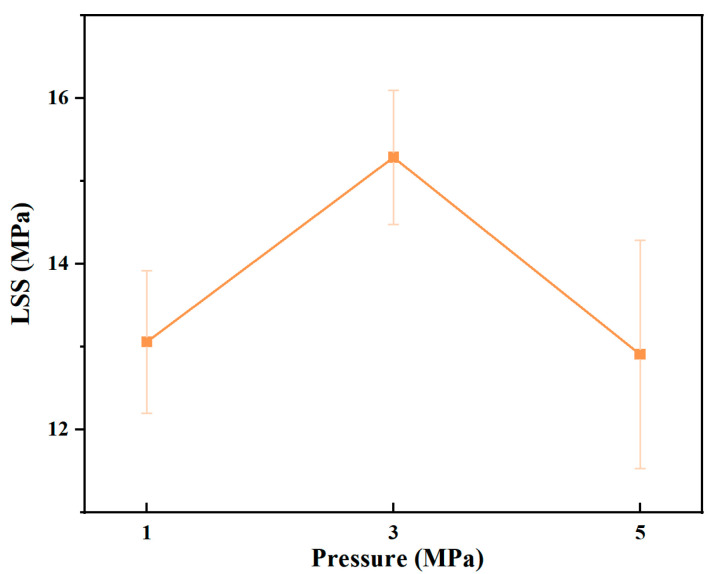
Strength of the hot pressing connection method.

Design Expert 13 was used to optimize and calculate the optimal process parameter range of the response surface model to achieve the desired LSS level. The top five best solutions are shown in Table 4. Within the scope of the plan, it is predicted that welded joints with an LSS of 13.73 ± 0.02 MPa can be obtained. The actual verification result is 13.58 ± 0.2 MPa, which is highly correlated with the numerical optimization solution, proving that BBD-RSM can effectively optimize for the actual welding situation. The optimized joint strength can reach 87.61% of the hot-pressing connection method (15.5 MPa). The strength of the hot pressing connection method is shown in Figure A1.

## 4. Conclusions

Among the various connection methods for thermoplastic composite materials, resistance welding has the characteristics of short processing time, simple surface treatment, high mechanical properties, simple equipment, and flexible operation. In an emergency, welding can be completed quickly using only resistive elements, a controlled power supply, and a clamping device. In the production process, the size of the welding area can be adjusted freely, and the heating efficiency of the resistance element can be flexibly controlled. Therefore, resistance welding is considered to be a very promising welding technique.

In this study, BBD-RSM was used to deeply explore the internal relationship between UCF/PPS welding strength and process factors. This method has the advantages of fewer tests, shorter calculation time, higher model accuracy, and accurate prediction results. It is hoped that it can be extended to other thermoplastic composite connection research.

Based on the analysis and discussion of the experimental results, the following conclusions are summarized:

(1) When the brass mesh size is 100 mesh, it has the best resin infiltration effect and the highest heating efficiency on the welding surface. After the 100-mesh brass mesh was oxidized, the surface roughness of the brass mesh was improved, and the interface bonding strength of the PPS resin was improved. This proves that a simple surface treatment can significantly improve welding strength. Finally, using Ox-RE/PPS for welding, the defects within the welding layer were reduced, and the welding strength reached 13.18 MPa. The failure mode changed from resin failure and implant tear to plate interlayer failure and carbon fiber fracture.
(2) The LSS of the joint first increased and then decreased with the extension of the holding time, and then tended to be stable, reaching a maximum value at 60 s. The single-factor experiment determined that the maximum welding strength was obtained at 310 °C, 1.15 MPa, and 120 kW/m^2^ for welding temperature, pressure, and power density, respectively. These three process factors were the main process factors in resistance welding.
(3) The parameters of the three main process factors were substituted into the BBD-RSM to construct a quadratic regression model with high fit and prediction ability. From the 3D surface diagram analysis, the influence of power density is the largest, and the interaction between welding temperature and power density is the most significant. Combined with the analysis of Design Expert 13 software, the optimal process parameters were obtained as follows: welding temperature: 313–314 °C, welding pressure: 1.04–1.2 MPa, power density: 124–128 kW/m^2^. After multiple factor optimization, the LSS of the welded joint prepared in the optimal parameter range reached 13.58 MPa, and the welding strength increased by 26.56% compared with the UT-RE without process optimization.
(4) Combined with the above conclusions, it can be concluded that BBD-RSM can effectively analyze and optimize the process parameters of resistance welding. Moreover, an efficient and systematic resistance welding process can obtain high-quality welded joints. However, due to the limited properties of the material itself, if we want to improve the resistance welding performance, we need to take other measures, such as resin layer reinforcement and interface bonding strength. For example, on the basis of resistance welding combined with other connection methods to achieve higher strength, surface modifiers (silane coupling agents, surfactants, etc.) are used to further enhance the interface properties between RE and resin, and fillers (short fiber materials, nanomaterials, etc.) are added to enhance the mechanical properties of the resin and then improve the joint properties.

## Figures and Tables

**Table 1:** The range of values of the three main parameters in BBD.

Level	Factor		
	A: T (°C)	B: F (MPa)	C: P (kW/m^2^)
−1	300	0.85	100
0	310	1.15	120
1	320	1.45	140

**Table 2:** Box–Behnken experimental design and results.

No.	A: T (°C)	B: F (MPa)	C: P (kW/m^2^)	LSS (MPa)
1	310	1.45	100	9.85
2	300	1.15	140	10.04
3	320	1.15	100	9.6
4	310	1.15	120	13.44
5	310	1.15	120	13.56
6	310	0.85	100	9.88
7	310	1.15	120	13.61
8	300	1.45	120	10.92
9	310	1.15	120	13.31
10	300	0.85	120	11.91
11	320	0.85	120	12.85
12	310	1.45	140	11.91
13	320	1.45	120	12.46
14	320	1.15	140	12.22
15	300	1.15	100	9.57
16	310	1.15	120	13.7
17	310	0.85	140	12.24

**Table 3:** The ANOVA results of the quadratic regression model.

Source	Sum of Squares	Degrees of freedom	Mean Square	F-Value	*p*-Value	Significance
Model	37.59	9	4.18	64.58	<0.0001	**
A	2.75	1	2.75	42.51	0.0003	**
B	0.3785	1	0.3785	5.85	0.0462	**
C	7.05	1	7.05	108.99	<0.0001	**
AB	0.09	1	0.09	1.39	0.2767	
AC	1.16	1	1.16	17.87	0.0039	**
BC	0.0225	1	0.0225	0.3478	0.5739	
A^2^	4.65	1	4.65	71.87	<0.0001	**
B^2^	0.8087	1	0.8087	12.5	0.0095	**
C^2^	18.85	1	18.85	291.38	<0.0001	**
Residual	0.4528	7	0.0647			
Lack of fit	0.3603	3	0.1201	5.19	0.0727	
Pure error	0.0925	4	0.0231			
R^2^	0.9881		Adjusted R^2^		0.9728	
Mean	11.83		Predicted R^2^		0.8447	
C.V. % ^a^	2.15		Adeq Precision		21.2956	

^a^ Coefficient of variation.

**Table 4:** The best solutions.

No.	A: T (°C)	B: F (MPa)	C: P (kW/m^2^)	LSS (MPa)
1	313.272	1.199	124.981	13.707
2	314.205	1.133	127.969	13.722
3	313.125	1.059	122.983	13.737
4	314.107	1.089	125.004	13.763
5	314.064	1.038	124.350	13.744

**Figure A2:**
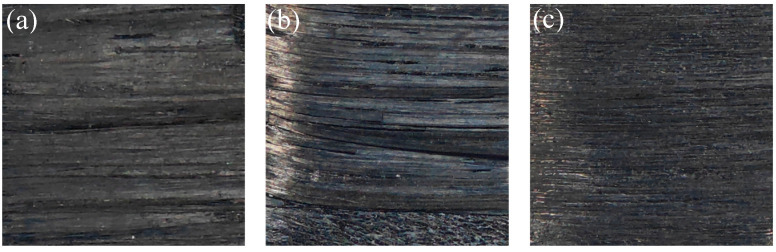
Macroscopic morphology of the failure surface: (**a**) 1 MPa, (**b**) 3 MPa, and (**c**) 5 MPa.

**Figure A3:**
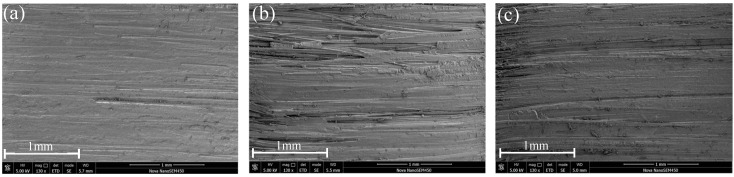
Microscopic morphology of the failure surface: (**a**) 1 MPa, (**b**) 3 MPa, and (**c**) 5 MPa.

## Data Availability

Data are contained within the article.

## Appendix A

The hot pressing connection method is the most basic one in hot-melting connection technology. In this section, the UCF/PPS splines were lapped together using the hot pressing process, and the resistance welding strength was evaluated based on the highest measured hot pressing connection strength. This experiment discusses the influence of different pressures on the hot-pressing connection effect when the hot-pressing die temperature is set near the processing temperature of PPS resin and there is sufficient melting and cooling time. The LSS test results of the hot-pressed connection method under different pressures are shown in Figure A1. The average strength of the joint formed at 3 MPa was 15.5 MPa. The maximum average LSS of hot-pressed specimens was used as a reference for evaluating the resistance welding strength. Figure A2 and Figure A3 show macroscopic and microscopic images of the failure surface.
